# The multiple roles of religious actors in advancing a sustainable future

**DOI:** 10.1007/s13280-025-02166-0

**Published:** 2025-04-05

**Authors:** Jens Koehrsen, Christopher D. Ives

**Affiliations:** 1 Centre for Religion, Economy, and Politics, University of Basel, Basel, Switzerland; 2 Theological Faculty, University of Oslo, Oslo, Norway; 3 School of Geography, University of Nottingham, Nottingham, UK

**Keywords:** Culture, Environment, Religion, Sustainability, Values, Worldviews

## Abstract

Religious actors have great potential for influencing transformation processes toward environmentally sustainable societies. Influencing peoples’ worldviews, values, and group norms, they can promote (or block) pro-environmental attitudes, lifestyles, and political decision-making. Yet, current scholarship is ambivalent about religion’s contribution to environmental sustainability. This perspective article outlines various roles religious actors can assume in sustainability transitions. We suggest a systematization of four roles—(1) pioneering, (2) path-following, (3) passive observing, and (4) prohibiting change—and portray five conditions that influence and catalyze these roles—(a) theological commitment, (b) internal support, (c) resources, (d) social and political influence, and (e) wider societal conditions. Generating this conceptual clarity is crucial as it allows researchers and policy actors to recognize the diversity of religious expressions with respect to sustainability action, and grasp the conditions under which religious actors are best equipped to address sustainability challenges.

## Introduction

Scholarship increasingly points to the importance of culture in the form of values, narratives, emotions, and worldviews for addressing sustainability challenges (Hulme 2020; Chapin et al. 2022; Richardson et al. 2022; O’Brien et al. 2023; Hochachka 2024; Varutti 2024). However, the particular role and significance of religion, which is deeply intertwined with human culture, has only recently begun to be appreciated in interdisciplinary sustainability literature (Otto et al. 2020; Smith et al. 2024; Stacey 2024). The ubiquity of religion (84% of the global population adhering to a religion; Pew Research Center 2012) and its impact on peoples’ worldviews and values positions it as a crucial factor in influencing transitions toward environmentally sustainable societies (Jenkins et al. 2018; Christie et al. 2019; Ives et al. 2020, 2023; Filimonau et al. 2022; IPCC 2022). Moreover, scholars have documented how religious actors wield their public voice and political networks to create awareness about environmental problems and to promote (or block) pro-environmental policies (Nadelman 2015; Schaefer 2016; Koehrsen 2021; Ives et al. 2023). Further, religious actors can use their resources (e.g., financial assets, infrastructures) to materialize sustainability transitions (e.g., renewable energy, energy-efficient building refurbishments, sustainable investments in stock markets, biodiversity in farming) (Mohamad et al. 2012; Amri 2014).

However, substantial debate remains in environmental literature over whether religions are indeed becoming "greener" and whether religion is an enabling influence or stubborn barrier to addressing environmental problems (Taylor et al. 2016; Wang et al. 2020; Michaels et al. 2021; Wilkins 2022; Djupe and Burge 2023). We contend that much of this debate stems from an under-recognition of the different roles that religious actors (e.g., religious leaders, religious organizations) can and do play in transitions toward environmental sustainability.

This perspective article outlines what role(s) religious actors can assume in transition processes. We place a focus on religious actors and their engagement with environmental dimensions of sustainability. Religious actors are defined here as discrete organizations or individuals that formally represent one or several religious traditions (Koehrsen 2015; Glaab 2017). They are, for instance, an imam, the Pope, a pastor, the Lutheran church of a specific country, a local Buddhist community, or an interfaith organization. We define religious traditions as belief systems that refer to some form of transcendence (e.g., supranatural entities such as gods) (see also Spiro 2013). Although focusing on religious actors in this article, on some occasions, we will use the term “religion” when referring to the broad phenomenon (e.g., religious beliefs, experiences, value systems). Further discussion of the definitional complexities of religion and religious actors can be found in Box 1.

This article proposes a systematization of the different roles of religious actors and discusses when they are likely to have an impact on these processes. This systematization enables researchers and practitioners (e.g., policy actors) to grasp under what conditions religious actors are best equipped to address sustainability challenges. Generating this conceptual clarity is crucial as it allows us to better understand under what conditions religious actors may become game changers in the ongoing transition processes. This knowledge can help practitioners to acknowledge, promote, and amplify religiously motivated environmental action. We use examples of religious environmental engagement from the literature and relevant cases from the Global North and Global South to support and characterize our typology. Moreover, we combine these with insights from established academic theory on social change for sustainability in order to create a framework (Scoones et al. 2015). In particular, we draw upon research on “sustainability transitions” (Loorbach et al. 2017; Köhler et al. 2019), defined as “long-term, multidimensional, and fundamental transformation processes” (Markard et al. 2012) by which societies become more sustainable in their lifestyles and modes of production and consumption. Besides technological and political change, transitions also involve cultural change in prevalent narratives, worldviews, and values (O’Brien 2018). As this perspective article will show, religious actors can assume impactful roles in these processes shaping consumptive processes and cultural conditions. Accordingly, religious actors may not only facilitate sustainability transitions, but importantly, also use their power to block and undermine these processes (Zaleha and Szasz 2015; Wexler 2016; Hayhoe et al. 2019; Veldman 2019).

Box 1: Complexities of defining religion The definition of religious actors is related to that of religion. There has been a broad debate in religious studies about the definition of religion. This debate has led to diverse approaches, some suggesting broader and others more limited definitions, while still others have argued for abandoning the term “religion” (Smith 1982; Aldridge 2007; Asad 2009; Woodhead 2011). Against the backdrop of this variety of approaches, the usual strategy of researchers has been to operate with working definitions of religion suited to the given academic project. Studying the relationship between sustainability and religion, religion could be perceived in four ways:

1. as a lens or methodological orientation that helps us to understand issues of sustainability in different ways without referring to groups that identify as religious (e.g., studying sustainability through the lens of rituals, see Stacey 2024);
2. as an implicit dimension of all sustainability action, since sustainability is fundamentally about values, norms, ethics. In this case, "sustainability" is seen as a pseudo-religious phenomena in itself (Johnston 2010, 2014);
3. as a discrete identity of groups, that can help us differentiate them from other actors (e.g., policy, industry, etc.) (Glaab 2017); and
4. as a dimension of individuals who may be acting for/against sustainability (i.e., their religious beliefs and practices) (Taylor 2010).

Focusing on religious actors, we opt for the third approach of perceiving religion. As such, we are placing an emphasis on a specific section (“actors”) within the broader phenomenon of “religion” (Taylor 2010; Stacey 2024). This approach allows us to distinguish religious actors from other types of actors such as businesses, political parties, universities. Yet, these other types of actors may sometimes involve religious dimensions (e.g., politician drawing upon religious beliefs in a public speech). At the same time, organizations that are usually perceived as “religious” often involve economic (e.g., financing) and political dimensions (e.g., hierarchies and decision-making). These overlaps create fuzziness when it comes to defining what actors count as religious ones. This question has been tackled in the debate about religious development organizations (often described as “faith-based organizations” or “religious NGOs”) (Koehrsen and Heuser 2020). In this debate, scholars have suggested that religious development organizations differ from other development organizations in their foundational philosophies which draw upon their specific faith basis, and thereby, refer to some form of transcendence (see also Berger 2003; Clarke 2006; Jennings and Clarke 2008). Employing faith basis as a criterion, they take into account that these organizations draw to different extents upon their faith basis, with some being “faith-centered” and others having a rather “passive” faith background (for different typologies of religious organizations according to the relevance of “faith” in them, see Sider and Unruh 2004; Clarke 2008; James 2009). Similarly, in this perspective article, we address actors with different levels of religiosity. Though we mostly focus on the more “faith-centered” actors (e.g., congregations, religious leaders), some of the examples also include organizations that may be perceived as religiopolitical ones (e.g., Evangelical think tanks). In the end, distinguishing religious actors from other types of actors allows us for studying their specific contributions to sustainability transitions. Only by making these conceptual distinctions between different types of actors we can make statements about their particular involvement in these processes.

## The relevance of religious resources for transitions toward sustainability

There are many historical examples of broader transformation processes to which religion has contributed. Religion has played a significant role in societal transitions, such as in the U.S. Civil-Rights-Movement and the Iranian and the Nicaraguan Revolution (Herbert 2002; Gardner 2003). Moreover, it is often considered as relevant for entrepreneurship and innovations (Keiper 2016; Yerxa 2016; Kumar et al. 2022). In particular, the broad membership basis and the influence of religious communities on membership behavior in the form of political protest, voting behavior, and daily practices (e.g., consumption habits) can facilitate change in society at large (Wald and Shye 1995; Lehrer 2004; Longest and Uecker 2018; Agarwala et al. 2019).

Religion as an enabling influence on social change is also evident in the current debates about religion and ecology. Contributions in these debates have highlighted the influence of religions on worldviews and values as a specific asset that religions can use to promote environmental sustainability (Gardner 2003, 2006; Holmes 2006; Tucker 2006; Gottlieb 2008; Bergmann 2009; Ives et al. 2024). Religious interventions into worldviews and values can lead to higher appreciation of nature, reduce anthropocentric beliefs, and improve the likelihood of pro-environmental behavior (Ives et al. 2023).

**Fig. 1:**
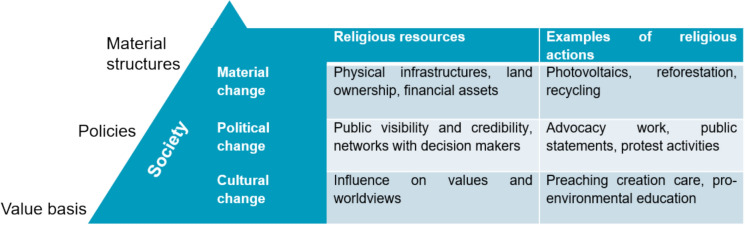
Religious intervention points for system change in society (based upon Koehrsen 2018)

Apart from cultural change, religious actors can draw upon their public visibility and credibility as well as their networks to promote political change (Nadelman 2015; Paullier 2017). Additionally, religious actors can also help to diffuse material change. Religious organizations can use their financial assets, physical infrastructure, and human resources to implement material changes in the form of energy-efficient refurbishments, reforestation projects, sustainable investment funds, and thereby stage this change for others (e.g., members) who may follow the example (Gardner 2002; Mohamad et al. 2012). Figure 1 summarizes the three most prominent ways in which religious actors may contribute to societal transition processes (Koehrsen 2015, 2018). The three ways correspond to different intervention points in society. This systematization resonates with broader debates in sustainability research about how system change can be initiated (Abson et al. 2017; O’Brien 2018; Dorninger et al. 2020; O’Brien et al. 2023). For each of type of intervention, religious actors can use specific resources listed in the figure.

A particularity of religion is its influence on culture. Although not unique to religion, religious actors are likely to have greater credibility with local communities regarding shaping beliefs and values than other actors (e.g., politicians, scientists). This has been long recognized in the field of development studies (Lunn 2009; Lipsky 2011; Clarke 2013; Heist and Cnaan 2016; Heuser and Koehrsen 2020). This ability of religion resonates with the rising focus on culture and deep social change in sustainability and climate change research (O’Brien 2018; Christie et al. 2019; Ives et al. 2020; Otto et al. 2020).

In total, religion can be vital for addressing sustainability challenges. However, religion is often neglected in the formulation and production of sustainability outcomes, in large part because of ignorance or confusion around the different roles that it can assume (Koehrsen 2018; Tobin et al. 2023; Stacey 2024). Understanding its different roles is crucial not only for researchers, but also for policy actors involved in designing governance strategies for sustainability processes.

## A framework for assessing the multiple roles of religious actors in transitions toward sustainability

The following framework systematizes four different roles of religious actors in transitions toward sustainability. We then build on this framework to clarify what conditions influence and catalyze these roles. This typology emerged through intensive dialogues between the authors in the context of an academic exchange program. The dialogues were informed by the authors’ longstanding knowledge of the research field, their reading of the literature, and their empirical research projects in the field. As this is a perspective article, it presents an original framework that can inspire future research which may help to support, revise, and further develop the heuristic.

### Four roles of religious actors

**Fig. 2:**
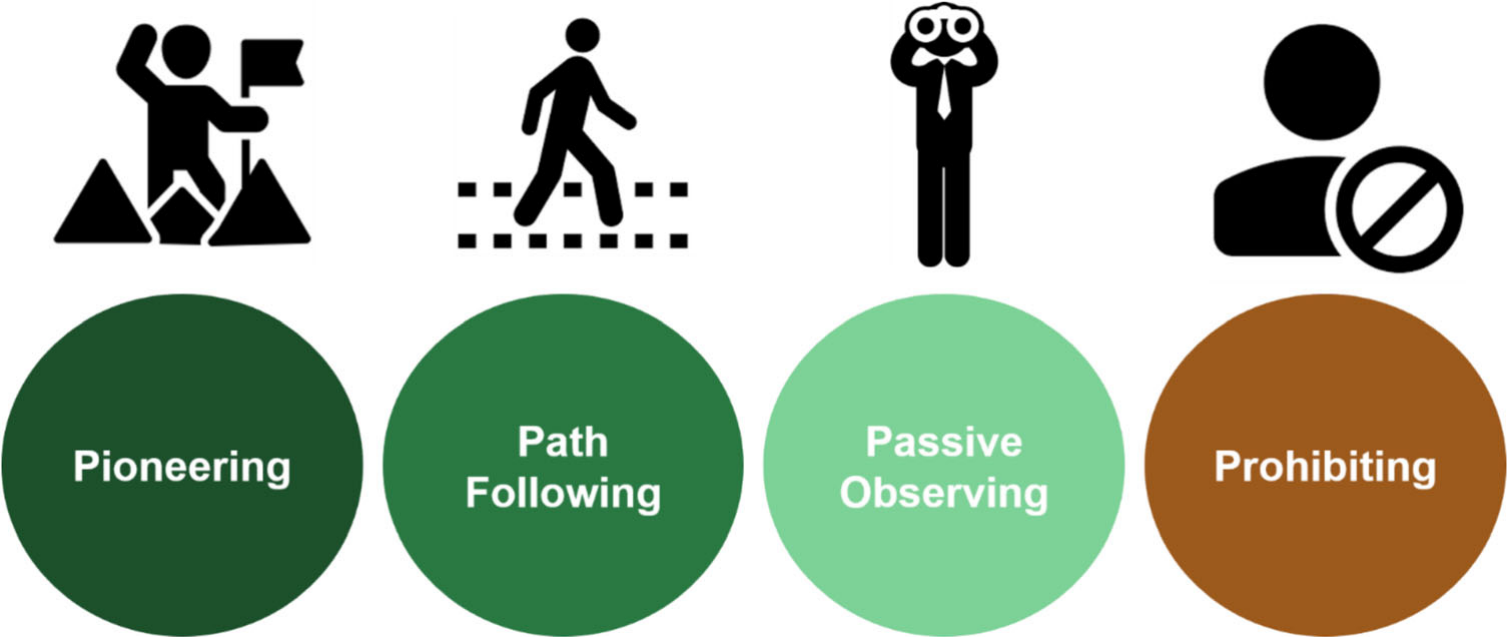
Four roles of religious actors in transitions toward sustainability

Religious actors can assume four ideal—typical roles (four P’s) in transitions (see Fig. 2): (1) pioneering, (2) path-following, (3) passive observing, or (4) prohibiting change. In order to avoid essentializing the roles (i.e., assuming that the actors are these roles), we use verbs to describe them instead of nouns (e.g., “pioneer”). This is because actors can assume a given role in a particular setting (e.g., a representative from a Muslim organization path-following change at a COP meeting) and another role in a different setting (e.g., the same representative pioneering or prohibiting change in the own local Muslim community).

This heuristic draws upon empirical observations about the different ways in which religious actors engage in sustainability transitions. While it does not claim to cover all possible roles, it includes the most relevant ones. In the following, we describe and illustrate each of these roles (see also Table 1).

**Table 1:** Four roles of religious actors in transitions

	Definitions	Illustrations
Pioneering	Initiating change	Introducing green theologies or practices (e.g., recycling) to a local community
Path Following	Adapting to ongoing change	Adopting new energy efficiency standards for religious buildings and societal trends regarding vegan food for religious events
Passive Observing	Not actively participating in change	No or few actions related to environmental sustainability beyond those legally required
Prohibiting	Blocking change	Supporting fossil-fuel industry in public statements, promoting overconsumption via preaching, voting against green measures in religious councils

*Pioneering actions* initiate change. Religious actors may do so alone or contribute together with other actors to initiating change. For instance, a Catholic priest, José Andrés Tamayo Cortez, spearheaded a protest movement against illegal logging in Honduras (Haynes 2007). In 2003, he led a 200-km march of 3000 people that created vast public awareness for the environmental problems in the country and inspired similar marches and environmental activities in Honduras. Religious actors will often be prone to assume a pioneering role when it comes to ethical issues (e.g., social justice related to environmental challenges; Kerber 2014, 2018; Chaplin 2016; Sexton and Pincetl 2022). In contrast, with regard to technological innovations or new sets of environmental behavior, religious actors may not necessarily be the ones that develop these in the first place. Nevertheless, they may be those that start to use them in a new setting (e.g., geographical region). In this sense, their pioneering role consists in being an early adopter of (technological) innovations. An example is the Catholic Church, which promotes the introduction of compost toilets in certain regions of Mexico (Valtierra Zamudio 2015). Another example is a Buddhist Foundation in Malaysia that provided a platform to experiment with recycling practices, thereby staging these practices and helping to diffuse them in the local social environment (Mohamad et al. 2012). As Mohamad et al. (2012) write this outcome was due to “the effective way in which religious vision and expectation are translated ‘practically’ within the religious aspirations of the community” (p. 244). In this perspective, religion’s ability to shape culture has been crucial for diffusing these new practices.

*Path-following* consists in adapting to the already ongoing change, following the trends in a certain setting (e.g., in a geographical region). For instance, religious organizations in Western Europe tend to perceive environmental engagement as an important societal trend that they need to follow suit in order to maintain their public image (Kidwell et al. 2018; Koehrsen and Huber 2021). Therefore, they increasingly address sustainability challenges by, for instance, undertaking measures to reduce CO2 emissions or taking up topics related to nature protection in religious services. Similarly, a survey in the US indicated that the dominant views on climate change in a certain state influence environmental positions of local congregations (Djupe and Olson 2010). Accordingly, religious organizations often adapt their environmental views to those of broader society. When pioneering action already established pathways for change, it becomes easier for religious actors to follow these and thereby upscale transition projects. For instance, a study on eco-congregations in Scotland shows that participating congregations found it comforting to be part of a wider movement of like-minded congregations (i.e., Eco-Congregation Scotland Network) (Kidwell et al. 2018). Another example is the Church of England’s commitment to “Net Zero” by 2030 (The Church Of England 2022). This plan was agreed in 2022. Although it is ambitious, with this plan the church has followed other charities and adopted the technical language of other types of organizations (e.g., businesses, environmental NGOs). Path-following can also become manifest in the change and dissemination of new values that then inform the lifestyle practices of religious followers. This is illustrated by followers of the Hare Krishna movement that have adopted a vegan diet despite the religion’s focus on dairy consumption (Lestar 2020). Broader debates in society about ethical farming and growing trends in veganism have led parts of the movement to reconsider its religious teachings and dietary practices.

When religious actors *passively observe*, they barely assume any active role in the transition process. For instance, studies on religious environmental action in Germany, Switzerland, and the United Kingdom show that many congregations (e.g., Christian, Muslim, Jewish, Buddhist, Hindu communities) undertake no or few actions related to environmental sustainability beyond those legally required (Koehrsen and Huber 2021; Harmannij 2023). Congregations often do not perceive environmental sustainability as a central topic given that their core tasks are others (e.g., providing spiritual service to members). Nevertheless, after passively observing the ongoing transition processes for a time, they may start to path-follow the change process by implementing small transition projects in the own religious community (Koehrsen et al. 2024). Alternatively, they may also begin to engage more actively by prohibiting change processes. Accordingly, the numerous passively observing religious communities constitute a kind of sleeping giant for transition processes that can potentially be mobilized into one or another direction.

*Prohibiting* of pro-environmental transitions includes actions that block transition efforts, question the existence of environmental issues (e.g., climate change skepticism), or promote lifestyles that aggravate environmental problems (Zaleha and Szasz 2015; Wexler 2016; Hayhoe et al. 2019; Veldman 2019). This may, for instance, happen when preachers of “prosperity gospel” disseminate religious narratives and values that encourage their followers to show God’s blessing through a lifestyle marked by overconsumption (e.g., regularly buying new clothes, driving big SUVs, overseas traveling) (Maxwell 1998; Meyer 2007; Comaroff 2009). Similarly, the evangelical think tank Cornwall Alliance illustrates a prohibiting role with its campaign “Resisting the Green Dragon” (Zaleha and Szasz 2014). This campaign published a DVD series with messages that sought to reverse “greening” processes among Evangelicals and in broader society in the US by portraying the environmental movement as “one of the greatest deceptions” (Chaplin 2016; Donovan 2017). A third example is evangelical actors that have supported the massive deforestation in Brazil (Otsuki 2013). Finally, also Muslim and Christian leaders in Sub-Saharan Africa will have prohibiting impacts on addressing sustainability challenges when publicly questioning the influence of humans on climate change and portraying its impacts (e.g., floodings, droughts) as God’s punishment for sinful behavior (Artur and Hilhorst 2012; Makame and Shackleton 2019; Nche 2023).

It is important to recognize that just like other sectors of society, diverse and varied actors' positions are to be expected in line with theories of social change. Sustainability transitions involve complex negotiation processes in which actors assume different roles (e.g., pioneering or prohibiting change) and may have good reasons for doing so. Indeed, failure to understand and appreciate some of the deeply held beliefs and worldviews that underpin prohibitive stances of religious actors can stymie effective action and partnership building. Each of the roles may perform a specific function in the transition process, contributing to the negotiations in different ways (e.g., prohibiting can lead to a more nuanced debate and developing solutions that are more acceptable for different stakeholders). Specific roles can become more relevant during certain moments of a transition process (Westley et al. 2013). While the interplay of pioneering and prohibiting can be important during the beginning of a transition process to negotiate the direction of change, path-following becomes crucial when a system is already reorganizing in a sustainable direction, and there is need to create a critical mass to help institutionalize the change. In particular, path-following could be relevant in the acceleration phase of transitions. The acceleration phase begins when innovations have been developed and start to diffuse more widely (Markard et al. 2020; Rosenbloom and Meadowcroft 2022). In this phase, religious actors could help to diffuse material, cultural, and political change more broadly into society.

What role an action assumes depends on the context in which it is situated. A given action may be pioneering within a religious community but path-following in the context of broader society. For instance, pioneering action in a religious community can mean that some of its members create an environmental group that starts initial projects in order to improve the energy efficiency of religious buildings. While this is pioneering action within the given religious community, it is path-following action for the broader society if other actors have already made energy efficiency an increasingly popular topic.

Moreover, religious actors can assume these roles on different socio-geographical scales (Kidwell 2020): (a) local, (b) national, and (c) global. At the local scale, a congregation may, for instance, assume a pioneering role by being the first actor to experiment with solar energy or recycling in a given municipality (Mohamad et al. 2012; Kidwell et al. 2018). Thereby, it introduces this innovation to the municipality and stages it to other actors who may consider adapting it. On the national scale, religious leaders and umbrella organizations can assume a pioneering role by mobilizing political protest, as the abovementioned example of a Catholic priest’s protest march against illegal logging in Honduras illustrates (Haynes 2007). On the international scale, COP meetings are a context in which religious actors frequently engage to undertake advocacy work for the most vulnerable (Kerber 2014, 2018; Glaab 2017). Here, together with other actors, they may be considered as pioneering communication about climate justice to expert committees and political decision-makers.

### Conditions that influence and catalyze roles

**Fig. 3:**
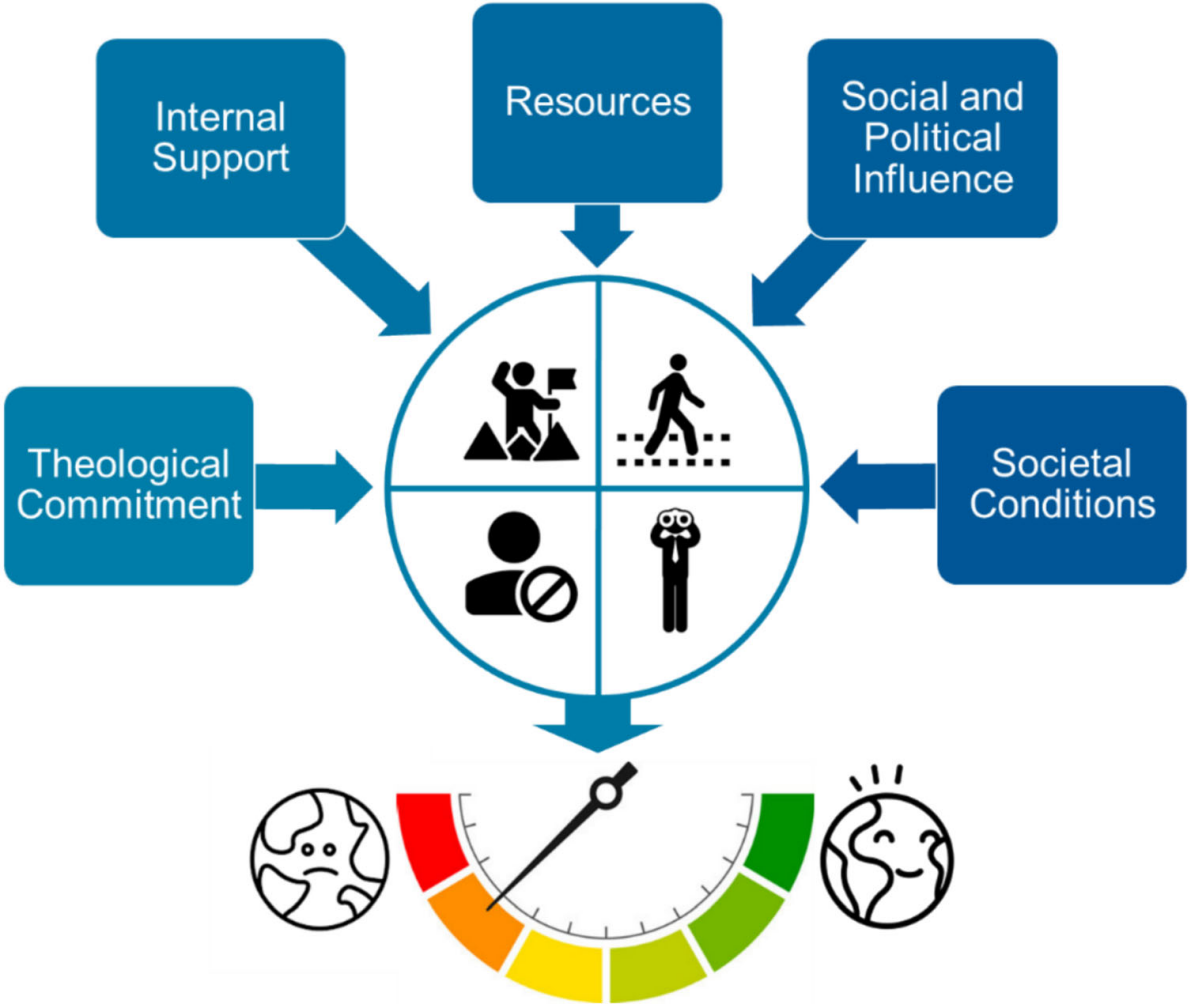
Condition domains that influence the role that religious actors will adopt, and how effective their actions will be at enabling or constraining environmental action

It is crucial to not only understand the various roles that different religious actors can adopt, but also the factors that determine why a given role is adopted and what impact it may have (for this, see also Berry 2022). Indeed, pioneering, path-following or prohibiting actions interact with smaller or bigger impacts in broader society, and societal conditions can strengthen the impact of these roles. Various internal and external conditions will influence a religious actor’s ability to effect change in broader society (Jackwerth-Rice et al. 2023). These are depicted in Fig. 3.

Below, five condition domains are outlined that can determine the role actors take and in turn the efficacy of their actions (Table 2): (a) theological commitment to environmental care and concern, (b) internal support that they enjoy from their communities, (c) resources that actors can deploy to effect change, (d) social and political influence within society, and (e) wider societal conditions that can open windows of opportunity or constrain actions.

**Table 2:** Supportive conditions for impactful action by religious actors

Conditions	Explanation
Theological commitment	The degree to which the theology and values of a religious community supports care and concern for environmental matters
Internal support	The transition action receives broad support within the given religious organization
Resources	The religious actor has a strong portfolio of resources (e.g., financial means, human capital, knowledge) that it can deploy to pursue change in society
Social and political influence	The religious actor has good relationship with the state and those with political power in society. This enables the actor to influence wider civic agendas
Societal conditions	Societal concern for environmental issues is a prerequisite for effective action, but religious actors will be influenced by whether or not such concern is being catalyzed by other actors (e.g., politicians, businesses, secular charities)

#### Theological commitment

Different religious groups will have different theological stances—beliefs and ideological positions related to a particular religious tradition—that shape how supportive they are likely to be of environmental action. These are often tied to narratives that justify the planned action. These can be, for instance, eco-theologies that call for action in the given transition domain (e.g., Islamic interpretation of humans as stewards of God’s creation; Khalid 2010; Dien 2013; Abdelzaher et al. 2019). Some adopt anthropocentric beliefs grounded in the exceptionalism of humanity over other living beings, while others are motivated by a belief in the sacredness of all the natural world or animistic spiritualities. Similarly, some religious expressions focus on future, "eternal" concerns or beliefs that climate change is a sign of apocalyptic "end times," while others emphasize duties and responsibilities toward justice in the present. Such beliefs are not neatly organized across religious traditions, with wildly different beliefs often present within the same tradition, denomination, or even particular community (Wardekker et al. 2009; Lawson and Miller 2011; Vincentnathan et al. 2016; Jamil 2023). Theological commitment to environmental care and concern will understandably shape which actor archetype an individual will adopt (e.g., pioneering vs. prohibiting). However, it is important to recognize that beliefs do not always align with behaviors (c.f. Gifford 2011), in that an espoused belief about the centrality of environmental stewardship can coexist with high-consumption lifestyles, or religiously affiliated environmental activists being motivated by other concerns than their religious faith. Moreover, beliefs alone will not determine the environmental action of religious organizations, as other factors—such as opportunity structures—are also important (Berry 2022). These will be discussed in the following.

#### Internal support

The degree of internal support within a religious community does not dictate whether or not a religious actor will adopt a pioneering, path-following or other role, but it will influence the kind of actions they will pursue and their efficacy in effecting broader change. An important prerequisite for pioneering transitions in broader society will be that there is internal support in the given religious organization that is pursuing the action, as internal tensions and conflicts would risk undermining the action and its impact. Research on sustainability transitions has shown that the likelihood of tensions and conflict rises as transition processes accelerate, gain in pace and start to exhibit potential structural changes (e.g., in infrastructures, laws, and lifestyles) (Markard et al. 2020). This becomes manifest within religious communities as well. Key actors or important sections of the membership within a given religion may resist initiatives to change the current state, thereby prohibiting pro-environmental transition efforts (Wilkins 2022; Koehrsen et al. 2023; Hearn et al. 2024). By contrast, extensive internal support facilitates societal change. This is illustrated by a case study about the role of the Catholic Church in preventing gold mining in El Salvador (Nadelman 2015): The anti-mining stance of the Catholic Church was supported by the entire Bishops Conference of El Salvador, which is usually marked by firm disagreements between liberal and conservative camps. This unanimity made it possible to gain wide public support for the anti-mining movement and to push the government to, first, temporarily stop mining, and finally in 2017, legislate a law to prohibit metal mining (Iglesias y Mineria 2017; Paullier 2017). This outcome is notable given that the extractive model for economic development has been popular among neighboring Latin American countries and that there was strong political interest in El Salvador to follow this model against the backdrop of extensive economic pressure. At the same time, the international mining industry undertook significant economic, legal, and political efforts as well as media campaigns to gain the support of politicians and voters. Therefore, at the beginnings of the 2000s, El Salvador seemed to join the pathway of extractivism. Nevertheless, by harnessing its high credibility and public visibility, the Catholic Church has been pivotal in inhibiting mining in El Salvador, thus illustrating the power of religious actors in addressing sustainability challenges.

#### Resources

The resources that a particular religious community has will enable or constrain the role of actors in taking a pioneering role for environmental sustainability. If a congregation or movement is financially constrained, there will be less willingness to focus on issues that are seen as outside core priorities (e.g., environmental sustainability) with energy directed toward financial health (Huber 2023). The material resources of a religious actor in the form of infrastructures and financial assets (e.g., money for public campaigning activities) will also have an influence on how effective actions or campaigns may be. A religious community’s expertise in a given transition domain will also have an impact on its ability to pursue change. Moreover, the level of religiosity in society—both the proportion of the population identifying with a religion and the strength of religious commitment—is also an essential condition for enacting change. The more active followers a religious community has, the more people can be reached by its messages and, potentially, be mobilized for political protest or voting and influenced in their worldviews, values, and lifestyles (Gardner 2003). For this, the extent of religiosity of the followers and their attachment to their religious community are important. For instance, in the case of a broad but mostly passive membership as in many Nordic countries (e.g., Church of Norway), the mobilizing effect is likely to be relatively low (Furseth et al. 2018), demonstrating how nominal religious affiliation is insufficient on its own.

#### Social and political influence

Of particular importance is political and social influence in the form of the state-religion relationship (Fox 2018a). If the relationship with the state and government actors is close, it enables the given religious actor to contribute to political legislation processes, and more easily adopt a pioneering role. For instance, in Indonesia, a national Muslim umbrella organization—Indonesian Ulema Council—and the Indonesian environment ministry collaborated to reinforce laws against illegal forest burning (BBC 2016). The umbrella organization supported the legislation by declaring the forest burning haram, forbidden by Islamic law (Mangunjaya and McKay 2012; Koehrsen 2021). By contrast, impactful religious action can be challenging if political actors are hostile toward religion (e.g., in the case of atheistic/anti-religious tendencies, see Dohe 2023). In these settings, religious actors may develop adaptive strategies by downplaying their religiosity (Glaab 2023; Krantz 2023).

Moreover, the extent to which a religious actor is perceived as trustworthy is a crucial symbolic resource, as it enables the actor to make credible contributions to public debates, raise public awareness, and thereby, create pressure on other actors (e.g., political decision-makers, business leaders). In general, these last conditions tend to be in place to a higher extent in many countries of the Global South (e.g., in sub-Saharan Africa, Latin America) than in most countries of the Global North (e.g., Western Europe) (Kerber 2018). For instance, in a recent survey in Latin America, 62.1% of respondents stated that they have some or a lot of trust in the Church, whereas all other public institutions, such as congress/parliament (20.5%), jurisdiction (25.7%), or police force (36.1%), score significantly lower in terms of trustworthiness (Latinobarómetro 2023). Accordingly, religious actors, such as the Catholic Church, remain the most trusted public institutions in Latin America, thereby having an advantage over other types of actors when it comes to making credible public statements.

#### Societal conditions

Finally, broader societal conditions can open windows of opportunity to facilitate impactful action in sustainability transitions (Geels 2002; Tongur and Engwall 2017; Geels et al. 2022). Windows of opportunity often emerge in the course of dramatic events (e.g., environmental disasters such as the Fukushima catastrophe) and with rising public pressure. These events lead to a questioning of the status quo (e.g., current carbon-based technologies) and may open the door for alternative trajectories (e.g., renewables) (Hermwille 2016). Windows of opportunity for religious action open up when there is high public concern in certain transition domains (e.g., climate justice) that is not taken up by other actors (e.g., politicians, businesses). Religious actors can adopt pioneering roles to fill the void by starting initiatives in these domains or by multiplying engagement (e.g., lobbying for climate justice). In doing so, they can harness the public concern and thereby mobilize other actors in broader society. An example is the abovementioned Catholic priest, José Andrés Tamayo Cortez, who created vast public awareness for the environmental problems in Honduras with his protest marches against illegal logging (Haynes 2007). By contrast, when engagement in transition activities has become a broad societal trend with high numbers of actors from different sectors engaging, the chances of having a visible religious impact seem to be lower. Nevertheless, there may still be opportunities when specific transition domains have received little attention (e.g., topics related to environmental justice). Such opportunity structures are often more relevant for religious engagement in sustainability transitions than the theological commitment, as Berry shows for the case of Evangelical climate denialism in the US and Pope Francis’ Laudato Si (Berry 2022).

#### Interactions and feedbacks

Complex social-ecological-technical systems are marked by feedbacks among system elements (e.g., actors, institutions, technologies) that can reinforce patterns of behavior and lock-in unsustainable trajectories (Folke et al. 2010; Moore et al. 2014; Williams et al. 2017). Such interactions are fundamental to "anthropocene traps" that have been identified at the planetary scale (Søgaard Jørgensen et al. 2024), and can influence religious actors’ roles. For instance, in Switzerland, religious NGOs and churches together with other actors spearheaded the responsible business initiative (The Federal Council 2022; Laruffa and Martinelli 2023). This initiative sought to establish a new law, rendering Swiss companies liable to Swiss prosecution for breaching human rights and environmental protection abroad. Voters narrowly rejected the initiative. Nevertheless, in the aftermath of the initiative, some politicians threatened to cut tax money for churches, as they viewed the initiative as an inappropriate religious intervention into politics. Moreover, the state financing for NGOs (including religious NGOs) was revised as a reaction to the initiative. Additionally, some church members who were business leaders and strongly against the initiative left their churches. As a result, the churches did not only lose members, but also the high financial contributions of these often wealthy members. These developments reduced the internal support for public sustainability campaigns within these religious organizations.

This example shows that religious action may result in feedback mechanisms that affect the resource basis and internal support. These feedbacks reduce or improve the chances for subsequent religious action. Therefore, anticipating potential feedbacks before taking action will be key for actions by religious actors to be effective (Moore et al. 2014). Having a rich portfolio of valuable resources (financial or otherwise) can render them reluctant to engage in transition endeavors, as responses by donors or other social actors may place these at risk (Fox 2018b). Conversely, such resources can increase organizational resilience and enable actors to continue to act even in the face of opposition. In contexts where religious leaders or organizations enjoy significant cultural respect or political influence, pioneering actions can initiate positive reinforcing feedbacks, whereby transition initiatives are amplified through normalization (scaling out) and moral association (scaling deep) (Lam et al. 2020). This is illustrated by the abovementioned example of the Catholic Church preventing gold mining in El Salvador. Public credibility and political influence enabled the church to amplify public concern about the environmental consequences of gold mining.

At the same time, path-following religious actors may perceive action for sustainability also as an opportunity to increase their resources, extending their membership and public credibility by engaging in a subject that has already become dominant in the wider sociocultural context (Koehrsen and Huber 2021). In this case, they will weigh what types of action will most likely improve their resource basis rather than endangering it.

## Outlook

There are similarities of religious actors with other actor groups, as well as unique differences. Thus, although this perspective has focused on religion, parts of the framework presented here can also be adopted to analyze the engagement of other types of actors beyond religious ones. Similar to other actors, religious actors can facilitate or block sustainability transitions in broader society. Yet in contrast with other actors, religious actors often enjoy a relatively high public credibility and have a strongly committed membership base that can be mobilized for or against societal transformations. Moreover, given their influence on peoples’ worldviews and values, they have advantages over other actors with regard to cultural change.

Religion seems to offer massive potential for transitions. Careful appreciation of the different roles religious actors can play will help sustainability practitioners to work more closely, sensitively and effectively with them. In particular, religious actors who, so far, passively observe transitions constitute a sleeping giant that could play a game-changing role. When “awakening the sleeping giant,” formerly passive religious actors can be, for instance, decisive in disseminating climate skepticism, as the case of the Christian right in the US shows (Veldman 2019). By contrast, if mobilized toward a path-following role, they could multiply and upscale existing transition initiatives and thereby accelerate ongoing change processes. However, it is unclear whether and to what extent this potential will be mobilized and how religious actors within pioneering, path-following and prohibiting roles will interact to accelerate or suppress action. In the face of accelerating climate breakdown and the associated socioeconomic polycrisis (Hoyer et al. 2023), will religions help provide stability and resilience in societies and support transitions to a sustainable future, or will they exacerbate instability and social unrest?

Geographical differences in religious identities, expressions, and positions in society will have crucial influence on likely outcomes. For instance, large Pentecostal churches (e.g., mega churches) in Latin America and sub-Saharan Africa could be well positioned to pursue change since they enjoy strong public visibility, have extensive numbers of highly committed members, and sometimes strong political influence (Kerber 2018). Yet, it is unclear what role they will assume, as their success builds in many cases on prosperity gospel, preaching a lifestyle of material abundance. Restricting the idea of economic prosperity could cause internal conflicts and decline in membership. Nevertheless, transition narratives that integrate ideas of prosperity gospel could help to gain internal support for environmental activities (e.g., concept of “green growth”).

Understanding the engagement of religion is a future research topic that sustainability researchers and policy actors urgently need to pay attention to. When governing transitions, it is necessary to grasp what role religious actors will assume, as they may support or block transition initiatives. They can be decisive opinion leaders that aggravate environmental problems by disseminating worldviews and values which promote the exploitation of natural resources (e.g., logging of rain forests, Otsuki 2013). Or, alternatively, they can help to address sustainability challenges by communicating values which inspire care for the natural environment (Ives et al. 2023).

Participatory dialogues within religious communities (c.f. Ives et al. 2024) could be a way to address potential tensions and foster internal support. Participation is time-intensive but can be a way to reach transitions that are more broadly supported and just (Skjølsvold and Coenen 2021; Newell et al. 2022). Integrating religious actors into policy and stakeholder dialogues could help improve understanding their standpoints and consider the roles that they assume when designing strategies to address sustainability challenges (Ives 2023). To some extent, this is taking place at the international level when the United Nations Environment Programme organizes events such as the “Global Faith Leaders Summit” (International Partnership on Religion and Sustainable Development 2023). Such events bring faith leaders together to discuss their role in addressing sustainability challenges, spur further commitment, and launch joint statements. However, these events mostly take religious actors into account that are already committed, while they are dominated by a small number of positional religious representatives. Thus, they remain restricted to the international scale, and their outputs (e.g., statements) often face difficulties in reaching the grassroots levels of the local faith constituencies (Veldman et al. 2014: 7). In order to reach the grassroots levels of religions, it is necessary to consider religious actors not only at the international or national, but also at the local scale. What is required are platforms of dialogue where representatives from municipal administrations and environmental programs could exchange with local religious actors around shared values. In this context, it is important to avoid an instrumentalization of religion (Jones and Petersen 2011). Local religious actors often focus on religious purposes (e.g., providing religious services to believers) and thereby will primarily follow their own goals that may not necessarily overlap (nor clash) with those of environmental sustainability.

## Conclusion

The role of religion in sustainability transitions has been under-recognized in sustainability debates, in part because of diverging perspectives on the degree to which faith-based actors may lead or support sustainability initiatives. By mapping out a spectrum of roles that religious actors may adopt, this article has provided a framework for research and practice that accounts for the variability observed.

The framework enables us to better comprehend why and how religious actors engage in transitions. In contrast to the common focus on the positive roles of religion (“greening of religions”), the framework also takes the passive and prohibiting roles of religious actors in sustainability transitions into account. Thereby, it avoids assumptions of homogeneity among actors that are in fact quite different, offering a way to break the deadlock of “greening” debates on religion and sustainability and enabling a more nuanced understanding of religious dynamics. Moreover, to guard against the risk of essentializing the roles, the framework makes allowance for the fact that the same religious actors may assume different roles in different contexts. Thus, it helps sustainability researchers and practitioners to understand the diverse and changing forms of religious engagement in sustainability transitions. In order to explain the changing roles and impact of religious engagement in these processes, the framework points out five conditions. Besides external opportunity structures, the internal dynamics within religious organizations (i.e., internal support and theological commitment) will be crucial for the ways in which religious organizations engage in transition processes. These internal dynamics often involve actors assuming different roles with some actors being passive while others endeavor to pioneer change, and again, others seek to prohibit it. In this way, the framework enables researchers to grasp the internal transition processes in religious organizations which can lead these organizations to become more active in the broader transition processes over time.

It is evident that religion will continue to have an enormous influence on societies globally and therefore we call for the sustainability science community to engage more deliberately with this topic in order to understand opportunities for religious mobilization of sustainability action, investigate dynamics that may accelerate change or lock-in opposition, and enable new forms of dialogue and exchange both within faith communities and among multi-stakeholder networks.
